# Pd Nanocatalyst Adorned on Magnetic Chitosan@*N*-Heterocyclic Carbene: Eco-Compatible Suzuki Cross-Coupling Reaction

**DOI:** 10.3390/molecules24173048

**Published:** 2019-08-22

**Authors:** Roya Sedghi, Bahareh Heidari, Hatef Shahmohamadi, Pourya Zarshenas, Rajender S. Varma

**Affiliations:** 1 Department of Polymer& Materials Chemistry, Faculty of Chemistry & Petroleum Sciences, Shahid Beheshti University, G.C, 1983969411 Tehran, Iran; 2 Regional Centre of Advanced Technologies and Materials, Department of Physical Chemistry, Faculty of Science, Palacky University, Šlechtitelů 27, 783 71 Olomouc, Czech Republic

**Keywords:** cross-linked chitosan, *N*-heterocyclic carbene, MWCNTs, Suzuki cross-coupling reaction

## Abstract

A novel magnetic-functionalized-multi-walled carbon nanotubes@chitosan *N*-heterocyclic carbene-palladium (M-f-MWCNTs@chitosan-NHC-Pd) nanocatalyst is developed in two steps. The first step entails the fabrication of a three-component cross-linking of chitosan utilizing the Debus–Radziszewski imidazole approach. The second step comprised the covalent grafting of prepared cross-linked chitosan to the outer walls of magnetically functionalized MWCNTs (M-f-MWCNTs) followed by introducing PdCl_2_ to generate the m-f-MWCNTs@cross-linked chitosan with a novel NHC ligand. The repeated units of the amino group in the chitosan polymer chain provide the synthesis of several imidazole units which also increase the number of Pd linkers thus leading to higher catalyst efficiency. The evaluation of catalytic activity was examined in the expeditious synthesis of biaryl compounds using the Suzuki cross-coupling reaction of various aryl halides and aryl boronic acids; ensuing results show the general applicability of nanocatalyst with superior conversion reaction yields, high turnover frequencies (TOFs) and turnover numbers (TON). Meanwhile, nanocatalyst showed admirable potential in reusability tests, being recycled for five runs without losing significant activities under optimum reaction conditions. The successfully synthesis of catalyst and its characterization was confirmed using the Fourier transform infrared spectrometer (FT-IR), spectrometer transmission electron microscopy (TEM), scanning electron microscopy (SEM), X-ray photo-electron spectroscopy (XPS) and thermogravimetric analysis (TGA).

## 1. Introduction

The Suzuki–Miyaura cross-coupling reaction plays a significant industrial scale role for the production of biaryl compounds which are widely used for a variety of industrial applications, such as the synthesis of natural products, herbicides, pharmaceuticals, polymers and agrochemicals [1,2,3,4]. Commonly, bulky phosphine ligands are employed to facilitate the Suzuki cross-coupling reaction; however, critical setbacks for this ligand are toxicity, air sensitivity and typical susceptibility to metal-ligand degradation [5,6,7], posing significant limitations on their uses [8]. In contrast, nitrogen-based ligands such as *N*-heterocyclic carbenes (NHCs) [9,10] have attracted attention as transition metal ligands; NHCs feature a significantly stronger electron-donating capacity than phosphine’s ligands, which increases the stability of resulting complexes towards the air, moisture and heat. On the other hand, the NHC ligands have the same σ-donor and low π-acceptor properties while they can form stronger bonds to a wide variety of metals such as Co [11], Pd [12], Cu [13] and Ni [14]. Pd complexes incorporating NHC appear to be a superior catalyst to achieve excellent catalytic activities in cross-coupling reactions. Also, the strong σ-donating properties of the NHCs promote oxidative addition of reagent and can assist in the reductive elimination of biaryl compounds [15,16]. On the other hand, recent advances in ‘green’ Suzuki cross-coupling reactions have focused on designing easily recoverable catalysts, thus simplifying product separation and adhering to sustainable green chemistry [17]. Although current homogeneous metal catalysts have sufficient activity, heterogeneous catalysts offer superior and practical aforementioned advantages. In recent years, the catalysts have been immobilized on a wide variety of supports such as Fe_3_O_4_ [18], SiO_2_ [19], TiO_2_ nanoparticles (NPs) [20] and mesoporous silica [21]; potential supports should have high stability and large surface area. The MWCNTs are robust support materials due to the high aspect ratio, thermal, chemical and mechanical stability, and conductivity and strong interactions for grafting to the other material with suitable appending groups [22]. In the last few years, techniques based on magnetic materials have attracted much attention to use in the wide variety of fields such as immunoassay [21], targeted drug delivery [23], and environmental detection [24]. The main advantage of magnetic material is to achieve easy separation strategy using an external magnetic field without additional centrifugation or filtration [25,26]. Also, due to the large surface area, and unique physical and chemical properties of the magnetic NPs, they have attracted much attention. In recent years biopolymers have gained a great research interest to use as a support for catalysts [27,28]. Chitosan is a naturally occurring linear copolymer that can be produced from chitin—the naturally occurring polysaccharide—via deacetylation [29]. Since the Chitosan is soluble in aqueous acidic media, it can be easily processed, and a wide variety of shapes are known including membranes, fibers or spheres [30,31,32]. The most important advantage of using chitosan is because of the large number of available amino groups in its structure which makes this polymer an ideal material for a wide variety of applications via modification of side groups that assist in cross-linking or grafting with other polymers [33,34]. On the other hand, imidazolium compounds are very versatile materials with a wide range of recent innovations in the field of chemistry; the design and synthesis of imidazole derivatives and imidazolium-containing polymers as a metal-ligand has received ample attention [35]. The Debus–Radziszewski imidazole synthesis strategy has been used in designing the cross-linked chitosan derivatives via a rather convenient one-pot approach involving the reaction of formaldehyde, a primary amine and a 1,2-dicarobonyl compound [36]. Akin to NHC ligand design, herein, the in situ interaction of imidazolium salt and the Pd source can be accomplished by deploying chitosan.

**Scheme 1:**
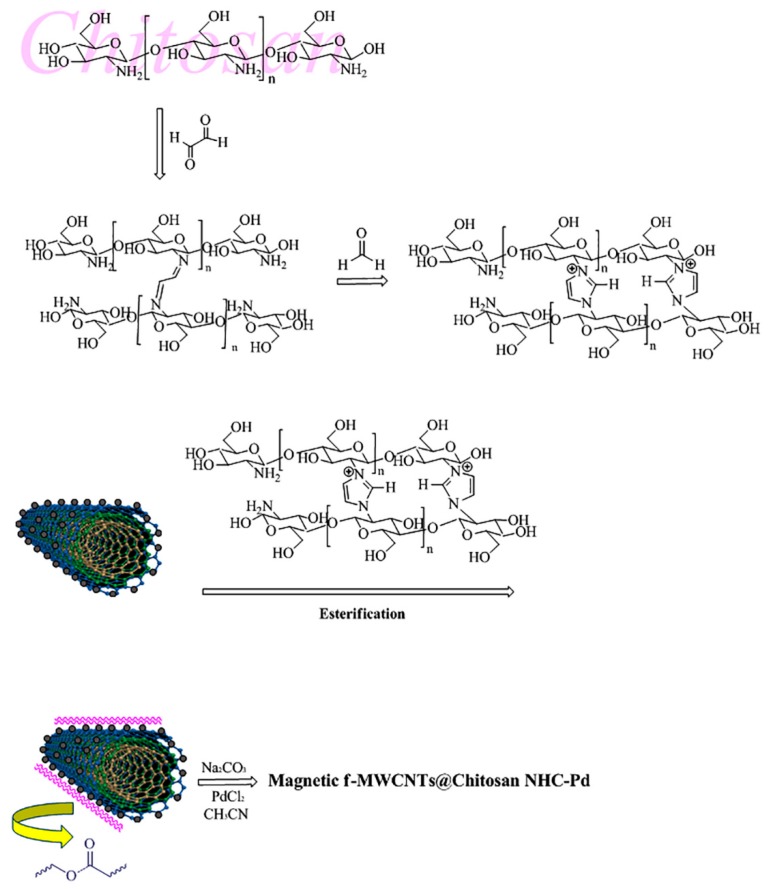
Schematic depiction of the preparation of magnetic-functionalized-multi-walled carbon nanotubes@chitosan (m-f-MWCNTs@chitosan) *N*-heterocyclic carbene-palladium (NHC-Pd) nanocomposite.

In this study, Debus–Radziszewski imidazole synthesis was used to fabricate cross-linked chitosan using glyoxal and formaldehyde as dicarbonyl and carbonyl components, respectively. The prepared cross-linked chitosan was anchored on the high surface area of m-f-MWCNTs via the esterification reaction between the hydroxyl group of chitosan and the carboxylic acid group from the surface of m-f-MWCNTs and then finally the prepared compound was applied to generate NHC@Pd (Scheme 1). To the best of our knowledge, combining MWCNTs and cross-linked chitosan provides not only a high surface area of MWCNTs but also enhanced the interaction of imidazolium cross-linked chitosan with Pd NPs which leads to an excellent opportunity for the design and application of this ideal combination.

## 2. Experimental

### 2.1. Reagents and Materials

MWCNTs, FeCl_3_.6H_2_O, FeCl_2_.4H_2_O, H_2_SO_4_ (98%), HNO_3_ (65%), NH_4_OH (28%), Glyoxal, formaldehyde, *N*,*N*’-Dicyclohexylcarbodiimide (DCC), 4-dimethylaminopyridine (DMAP), palladium chloride (PdCl_2_), dimethyl sulfoxide (DMSO), dimethylformamide (DMF), acetonitrile (CH_3_CN), ethyl acetate, n-hexane and ethanol were purchased from Merck Company (Darmstadt, Germany). Chitosan, boronic acids and differently substituted aryl halides were purchased from Sigma-Aldrich Company (St. Louis, MO, USA). All employed solvents were dried using standard process and freshly distilled prior to use.

### 2.2. Instrumentation

The Fourier transform infrared (FT-IR) measurements were performed using a FT-IR spectrometer (BOMEM MB-Series, Quebec City, QC, Canada) in the form of KBr pellets over the wavenumbers of 400–4000 cm^−1^. The thermal properties of the nanocomposites were investigated using thermal analyzer instrument (TGA/DTA BAHR: STA 503, Hüllhorst, Germany) from 25 to 1000 °C at a heating rate of 10 °C/min under air. For determination of Pd loaded on nanocomposite an atomic absorption spectrometry (Shimadzu model AA-680, Kyoto, Japan) was used with air-acetylene flame atomizer and equipped with hollow cathode lamp. The SEM (Philips XL30, Leuven, Belgium) was used for the investigation of surface morphological features and size of nanocatalyst and in order to provide more quantitative data of surface, image of nanocatalyst was taken using TEM (Philips CM30, Milan, Italy). The X-ray photoelectron analysis (XPS) was performed using a thermo fisher scientific k-alpha instrument (Waltham, MA, USA). The Brunauer–Emmett–Teller (BET) surface area was determined using a BET analyzer (Belsorp mini, Osaka, Japan). The progress of the Suzuki cross-coupling reaction was monitored by gas chromatography (Shimadzu model GC instrument, Kyoto, Japan). 

### 2.3. Synthesis of Functionalized-MWCNTs (f-MWCNTs)

In order to improve the dispersion and reduce the aggregation tendency of MWCNTs and also for improved interaction of cross-linked chitosan with surface of MWCNTs, surface functionalization of MWCNTs (carboxylation) was carried out. According to the previously reported procedure [37], 2 g MWCNTs were ultrasonically treated in a 100 mL of HNO_3_/H_2_SO_4_ (1:3) mixture at 60 °C. After 2 h stirring, the resulting product (f-MWCNTs) was collected and washed with distilled water and then dried at 60 °C for 24 h.

### 2.4. Synthesis of Magnetic f-MWCNTs (m-f-MWCNTs)

Firstly, 1 g of obtained f-MWCNTs nanocomposite was dispersed in 110 mL distilled water and then 0.8 g FeCl_3_.6H_2_O was added to the reaction mixture under vigorous stirring and under nitrogen atmosphere. The solution was stirred for 5 min and followed by addition of 1.2 g FeCl_2_.4H_2_O. Then 50 mL diluted NH_4_OH was added drop-wise to the reaction mixture and was kept at 60 °C for 3 h. The final products were isolated using a permanent magnetic field and washed several times with distilled water and ethanol. The obtained m-f-MWCNTs nanocomposite was dried at 25 °C for 24 h.

### 2.5. Synthesis of the Cross-Linked Chitosan Using Imidazole

Briefly, 1 g of medium molecular weight chitosan (6.1 mmol) was dissolved in 100 mL diluted CH_3_COOH (concentration 10%), then 0.35 mL (3.1 mmol) of glyoxal (40% in water) and 0.114 mL (3.1 mmol) formaldehyde were added to chitosan solution while it was being stirred vigorously. After attaining the homogeneous solution, a required amount of diluted NaOH (concentration 10%) was added drop-wise till the pH reached 5. The ensuing solution kept under constant stirring over 48 h at room temperature.

### 2.6. Substitution Cross-Linked Chitosan with Activated m-f-MWCNTs (Esterification)

The cross-linked chitosan was covalently linked onto the m-f-MWCNTs via a chemical reaction between the hydroxyl groups of the cross-linked chitosan and the active carboxyl groups of m-f-MWCNTs. To accomplish the purpose, 0.1 g of m-f-MWCNTs and 0.16 mL of DCC and 0.8 g DMAP were added to DMF (20 mL) while the mixture was stirred at room temperature for 30 min, followed by the addition of 0.5 g of cross-linked chitosan. The reaction mixture was stirred for 48 h and then the resulted product was rinsed with distilled water (3 times) and dried under vacuum for 24 h at 25 °C.

### 2.7. Preparation of the m-f-MWCNTs@chitosan NHC-Pd

In order to increase the solubility of the PdCl_2_ in aqueous solution, CH_3_CN was added to solubilize the complex. M-f-MWCNTs@cross-linked chitosan (0.2 g) was added to a solution of PdCl_2_ (MeCN)_2_ (5 mL) in DMF (10 mL) and the reaction mixture was stirred for 24 h at 60 °C. The final precipitated solid was collected using filtration under vacuum and washed with ethanol and distilled water to remove any adsorbed Pd from the surface of the composite.

### 2.8. General Procedure for the Suzuki Reactions

To establish the generality of this nanocatalyst, a wide variety of aryl halides and arylboronic acids were used. In a typical experiment, to a 50 mL flask an appropriate aryl halide (1 mmol), arylboronic acid (1.2 mmol), K_2_CO_3_ (3 mmol), the above-mentioned catalyst (0.1–1 mol% based on aryl halide) and 6 mL EtOH/water (1:1 *V*/*V*) were successively added. The mixture was stirred vigorously for the varying particular length of time (2–8 h) and temperature at (40 °C–60 °C) depending on the substrate used. Upon completion of the reaction, the mixture was cooled down to ambient temperature (25 °C) and the solid catalyst was separated by using a magnet bar. The filtrate was extracted three times with organic solvent (ethyl acetate). Then the organic phase was dried over anhydrous MgSO_4_ and after removal of the solvent, corresponding cross-coupled Suzuki products were purified by short chromatography on a silica gel (n-hexane/ethyl acetate) to afford the desired product and calculate the yield.

## 3. Result and Discussion

Homogenous Pd NPs have been extensively employed in various Pd catalyzed cross-coupling reactions such as Suzuki [38], Sonogashira [39], Heck [40] and Negishi [41]. The inherent tendency toward NPs agglomeration makes the use of supporting agents or stabilizers obligatory in most reactions. The main purpose of this study has been to develop robust Pd NPs stabilized onto a novel supported polymer which is abundant, renewable and biodegradable; such a decorated Pd nanocatalyst on m-f-MWCNTs with larger surface area coupled with ease of its separation using just an external magnet bodes well for its numerous Pd-catalyzed reactions.

### 3.1. Characterization of Nanocatalyst

#### 3.1.1. FT-IR Spectroscopy

**Figure 1:**
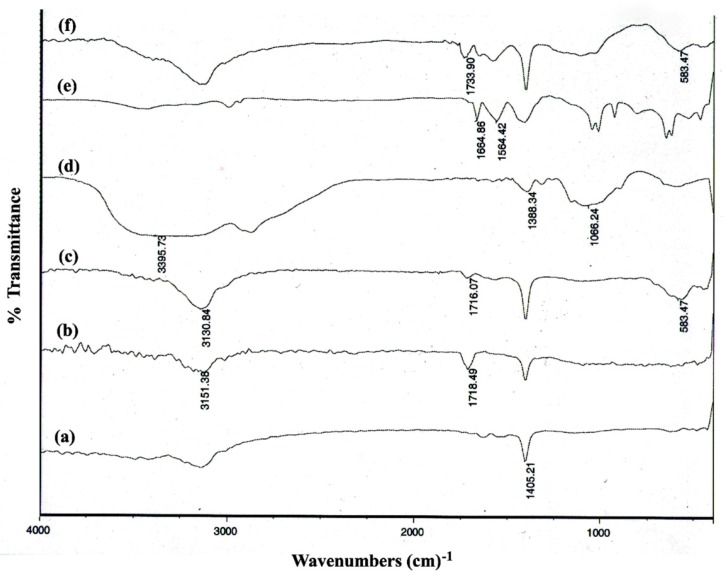
FT-IR spectra of (**a**) multi-walled carbon nanotubes (MWCNTs), (**b**) functionalized-MWCNTs (f-MWCNTs), (**c**) magnetic f-MWCNTs (m-f-MWCNTs), (**d**) chitosan, (**e**) cross-linked chitosan, and (**f**) m-f-MWCNTs@cross-linked chitosan.

Figure 1 shows the FT-IR spectra for (a) MWCNTs, (b) f-MWCNTs, (c) m-f-MWCNTs, (d) chitosan, (e) cross-linked chitosan, and (f) m-f-MWCNTs@cross-linked chitosan. In Figure 1b, absorbance peaks at 1718 cm^−1^ and 3151 cm^−1^ were attributed to stretching bands vibration of C=O and O–H, respectively that confirmed successful carboxylation of MWCNTs (Figure 1a) [42,43]. For m-f-MWCNTs (Figure 1c), the band at 583 cm^−1^ is attributable to Fe–O stretching vibration. In the spectrum of cross-linked chitosan (Figure 1e), the appearance of the new band at 1564 cm^_1^ and 1664 cm^_1^ were accredited to the aromatic C=C stretching and C=N ring stretching vibrations, respectively, due to the formation of an imidazole ring [44,45]. The successful esterification reaction between OH groups of chitosan and carboxyl groups emanating from m-f-MWCNTs was confirmed by observation of 1733 cm^−1^ band (Figure 1f) [46].

#### 3.1.2. XPS Analysis

**Figure 2:**
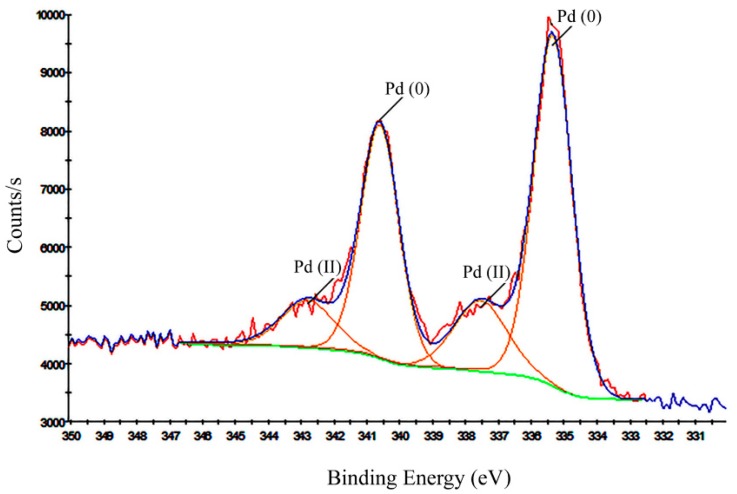
XPS of the m-f-MWCNTs@ chitosan NHC-Pd nanocomposite.

XPS spectroscopy was applied to investigate the electronic state of Pd species that exist within the nanocatalyst. As shown in Figure 2, two peaks were centered at 335.48 eV and 340.94 eV which are ascribed to Pd^0^ 3d_5/2_ and 3d_3/2_, respectively, and two peaks were centered at 338.2 and 343.5 eV which accredited to Pd (II) 3d_5/2_ and 3d_3/2_, respectively [47]. These observations are reminiscent of previous literature reports [48,49], wherein parts of Pd (II) species were likely reduced by chitosan backbone.

#### 3.1.3. TGA Analysis

**Figure 3:**
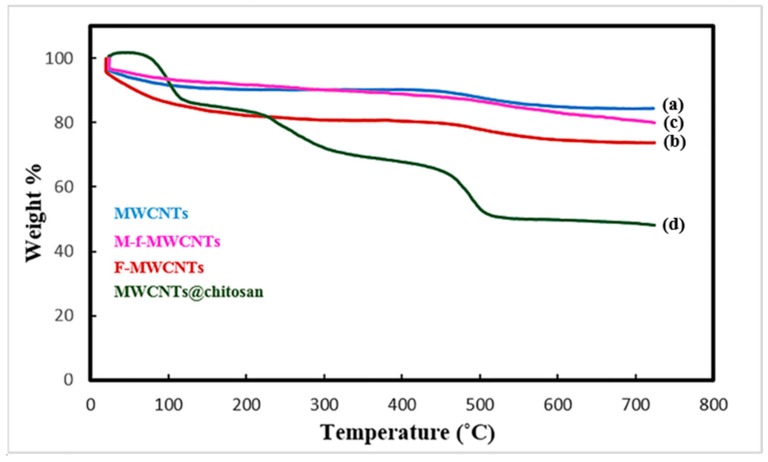
TGA plots of (**a**) MWCNTs, (**b**) f-MWCNTs, (**c**) m-f-MWCNT, (**d**) m-f-MWCNTs@cross-linked.

Thermograms provide useful information about the catalyst contents in the nanocomposite powder (Figure 3). It is well known that the graphitic structure of MWCNTs is stable up to 600–700 °C. As can be seen, decomposition of f-MWCNTs with strong acids (carboxylic acid groups at the surface of nanotube) occurs gradually in the whole range of temperature. Results showed that f-MWCNTs lost 27 wt.% by 700 °C while m-f-MWCNTs is thermally stable beyond this and lost 19 wt.% by 700 °C. The trend of weight loss for m-f-MWCNTs@chitosan is moderate and faster than of other samples up to 500 °C due to the decomposition of polymeric portions of the nanocomposite.

#### 3.1.4. SEM and TEM Images and EDS Analysis

**Figure 4:**
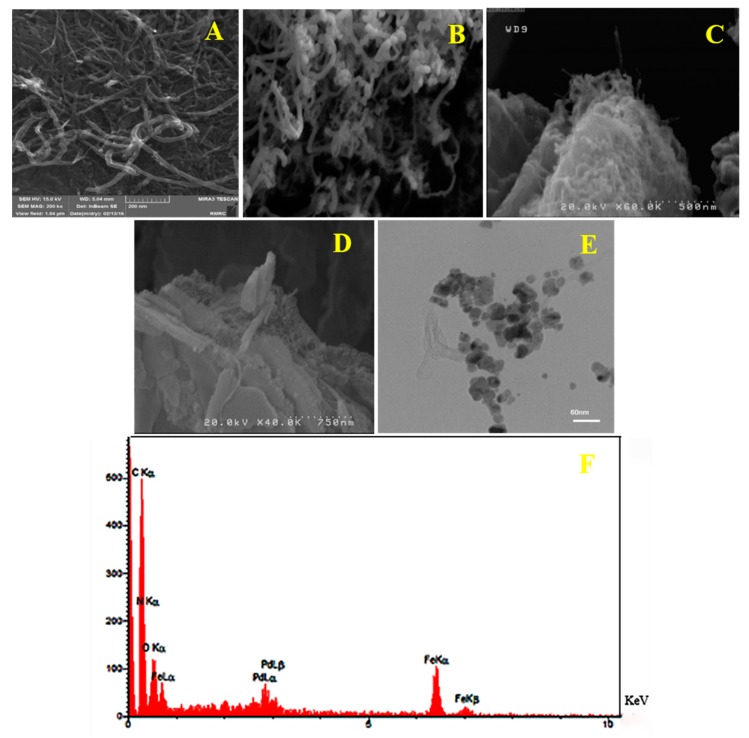
SEM images for (**A**) f-MWCNTs, (**B**) m-f-MWCNT, (**C**) m-f-MWCNTs@chitosan NHC-Pd nanocomposite, (**D**) m-f-MWCNTs@chitosan NHC-Pd nanocomposite after five runs, (**E**) TEM image for m-f-MWCNTs@chitosan NHC-Pd nanocomposite and (**F**) the EDX spectrum of m-f-MWCNTs@chitosan NHC-Pd nanocomposite.

The morphological feature of the products before and after the assembly of imidazolium cross-linked chitosan to m-f-MWCNTs and dimensions of the MWCNTs, m-f-MWCNTs and m-f-MWCNTs@chitosan NHC-Pd were recorded using SEM (Figure 4). The surfaces of MWCNTs are smooth and tidy (Figure 4A), whereas m-f-MWCNTs (Figure 4B) have a spherical shape and smooth surface morphology. In contrast, the m-f-MWCNTs@chitosan NHC-Pd surfaces (Figure 4C) become rough and they have many new branches. Compared to MWCNTs, the m-f-MWCNTs@ chitosan NHC-Pd nanocomposite has a more aggregate morphology which possibly is due to the interactions among the functional groups of cross-linked chitosan and the external wall of m-f-MWCNTs and thereby affirming that cross-linked chitosan is successfully attached to m-f-MWCNTs surfaces. It is further substantiated by FT-IR and TGA characterizations. Figure 4D shows a SEM image of the recycled m-f-MWCNTs@ chitosan NHC-Pd nanocomposite. The morphology of m-f-MWCNTs@chitosan NHC-Pd was examined by TEM, the result is shown in Figure 4E; apparently, the modification of surface m-f-MWCNTs has occurred successfully, suggesting that the cross-linked chitosan was coated onto the MWCNTs. The obtained data for EDX analysis affirmed the presence of Pd NPs in the catalyst structure (Figure 4F).

#### 3.1.5. BET Analysis

The BET surface areas of the MWCNTs and the catalysts are provided. After modification of the MWCNTs, the BET surface area decreased dramatically from 430.06 m^2^ g^−1^ in MWCNTs to 283.85 m^2^ g^−1^ in the final nanocatalyst; the obtained result indicated that most of the external surface of the MWCNTs was impregnated with the Pd.

### 3.2. Catalytic Performance of m-f-MWCNTs@chitosan NHC-Pd Nanocomposite

Heterogeneous catalysts stabilized by polymers have been extensively investigated for their cross-coupling reactions [50,51]. However, there are only a few reports on the application of magnetically separable nanoparticles stabilized by hydrophilic polymers which catalyze the cross-coupling reactions under greener conditions [25,52,53,54,55,56]. In this work, the catalytic activity of Pd NPs stabilized on m-f-MWCNTs@cross-linked chitosan nanocomposite was investigated for the Suzuki reaction under eco-friendly conditions. The flexibility in surface functionalities, large surface area to volume ratio and superior mechanical performance of m-f-MWCNTs positively influenced the amount of catalyst loaded and decreased the required reaction times. In this work, the reaction of bromobenzene with phenyl boronic acid in the presence of the aforementioned catalyst was studied as a model reaction (Table 1). According to the obtained data, the best result was found using a mixture of EtOH:H_2_O as a solvent of choice. The use of this mixture as the solvent provides excellent interaction between heterogeneous stabilized Pd nanocomposite with hydrophilic compound (boronic acid) and hydrophobic compound (aryl halides) which consequently leads to an increase in the yield and rate enhancement of the Suzuki reaction. According to the previous experiments—the base significantly affected the product yields in this regard—K_2_CO_3_ was used in a catalytic system. The amount of catalyst used and the time of the reaction are two significant parameters investigated in this work. The best conversion in the model reaction to provide biphenyl was identified by the deployment of 0.1 mol% of catalyst in 3 h for completion of the reaction (Table 1). As shown in Table 2, the coupling products obtained from the reaction of boronic acid derivatives with various aryl halides gave corresponding biaryls (entries 1–18). As shown, the iodobenzene, 4-iodotoluene and 4-iodoanisole gave the corresponding biaryls in the presence of 0.1 mol% catalyst in only 2 hrs (Table 2, entries 1–3). Both electron-donating and -withdrawing groups on aryl bromides gave the corresponding biaryls in excellent yields in the presence of 0.1 mol% catalyst in only 3 h (Table 2, entries 4–6). The prepared catalyst efficiently catalyzed the Suzuki cross-coupling reaction of aryl iodides and aryl bromides. It has been well-established that Suzuki reaction does not proceed smoothly when aryl chlorides are used; aryl chlorides are commonly less reactive since C–Cl bond is stronger than those of C–Br and C–I, thus requiring longer reaction times, and catalysts with higher activity are a prerequisite. Because of the ready availability of aryl chlorides and being relatively less expensive than their bromo and iodo counterparts, using them in cross-coupling reactions, including Suzuki reaction, is desirable. Thus, the most important advantage of our novel catalyst system is that it can be successfully used in Suzuki reaction wherein a wide variety of aryl chlorides can be coupled with aryl boronic acid to generate the corresponding products. However, aryl chlorides due to strong C–Cl bond, relative to C–Br and C–I, are more difficult to react than aryl bromides and aryl iodides and, therefore, increased amounts of catalyst and longer reaction time are often required. By increasing the amount of catalyst loading to 1 mol% and extending the reaction time to 8 h in 60 °C (entries 8–10 and 16–18), the Suzuki reaction of aryl chlorides could be completed in good yields (40–62%). Furthermore, 4-tolylboronic acid was subjected to various aryl halides and the obtained data are documented in entries 11–18.

### 3.3. Study of Pd Leaching

The hot filtration test was performed to investigate whether the reaction proceeded in a heterogeneous or homogeneous mode. In a typical experiment, phenyl boronic acid (1.2 mmol), bromobenzene (1 mmol), K_2_CO_3_ (3 mmol), m-f-MWCNTs@chitosan NHC-Pd nanocomposite as catalyst (0.1 mol%) were taken in solvent (H_2_O-EtOH: 1:1 (6 mL)) in a round-bottomed flask and stirred at 50 °C for 30 min. After 30 min of reaction, the hot reaction solution was separated by a magnet and the experiment was continued for another 3 h. According to the obtained data (monitored by GC), no further progress in the Suzuki cross-coupling reaction was observed even after a 3 h reaction time. Thus, barely any Pd leached into the reaction mixture, which confirmed that the reaction proceeded in a heterogeneous fashion.

According to the literature review [57] and as evidenced by local leaching studies, the following two essential factors have emerged: (1) the morphology of a heterogeneous catalyst is retained and (2) a very low level of metal leached in to the solution phase, thus implying that the catalyst system is a ‘cocktail’-type. However, for our prepared composite structure, where Pd NPs were dispersed on this composite structure, we cannot assign the Pd NPs shapes, but the hot filtration test confirmed the absence of leached Pd in solution.

### 3.4. The Reusability and Stability of Nanocatalyst

**Figure 5:**
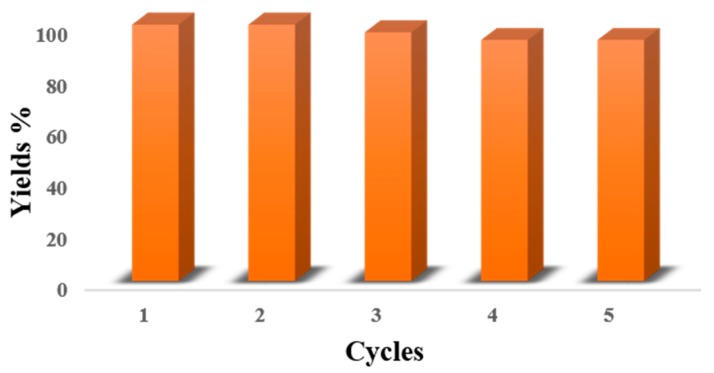
Reusability studies for recycling of m-f-MWCNTs@chitosan NHC-Pd nanocomposite catalyst in Suzuki reaction of bromobenzene with phenyl boronic acid.

The reusability of the nanocatalyst was studied in the coupling reaction of bromobenzene and phenyl boronic acid as a model reaction under the identified optimal conditions. In each cycle, 6 mL aqueous solution containing reagents and the nanocatalyst was added into a 50 mL round-bottomed flask at optimal temperature. Upon the completion of the reaction, nanocatalyst was separated from the mixture using an external magnetic field, washed with water and reused for the next cycle in the same reaction. The results showed that the nanocatalyst could be successfully reused for five runs, without any considerable loss in its catalytic activity for the Suzuki cross-coupling reaction (Figure 5). The remaining amount of Pd after the fifth cycle was decreased by 0.325 mmol g^−1^ to 0.308 mmol g^−1^ as detected by AAS analysis (5% leaching).

### 3.5. Comparison

To evaluate the efficiency of the m-f-MWCNTs@ chitosan NHC-Pd nanocomposite, and its catalytic prowess, the activity was compared to similar earlier documented reactions from the literature. According to the results summarized in Table 3, the reduced time reaction, high catalytic efficiency under greener media at reduced reaction temperature, higher conversion and yield were obtained by using our catalyst [58,59,60,61,62,63,64].

## 4. Conclusions

Our investigations demonstrate that the immobilization of imidazolium cross-linked chitosan to m-f-MWCNTs support, followed by decorating with palladium, produces a well-defined and superior catalyst for the Suzuki cross-coupling reactions. The repeated amino groups of chitosan led to imidazole units’ in polymer chains that enhanced the number of Pd linkers and catalyst efficiency. Our best prototype catalyst demonstrated an encouraging range of catalytic activity in the Suzuki cross-coupling reactions of aryl chlorides in aqueous media. Further, this catalyst can be easily separated by using a magnetic field and used for five runs without losing significant activities under optimum reaction conditions; high TOF and TON, two important green chemistry metrics, bodes well for its utility in other related Pd-catalyzed reactions.

## Figures and Tables

**Table 1:**
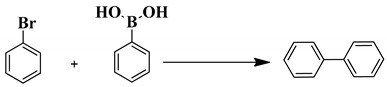
Optimization of the reaction conditions for the Suzuki reaction of bromobenzene with phenyl boronic acid catalyzed by m-f-MWCNTs@chitosan NHC-Pd nanocomposite.

Entry	Amount of Catalyst (mol%)	T (°C)	Solvent	Time (h)	Conversion (%)	Yield (%)
1	0.1	80	H_2_O	5	80	75
2	0.5	80	H_2_O	5	90	84
3	0.5	80	H_2_O-EtOH	5	100	97
4	0.3	80	H_2_O-EtOH	5	98	94
5	0.1	80	H_2_O-EtOH	5	97	94
**6**	**0.1**	**50**	**H_2_O-EtOH**	**3**	**97**	**94**
7	0.08	50	H_2_O-EtOH	3	85	80

Reaction conditions: Phenyl boronic acid (1.2 mmol), bromobenzene (1 mmol), K_2_CO_3_ (3 mmol), m-f-MWCNTs@chitosan NHC-Pd nanocomposite as a catalyst, Solvent (H_2_O-EtOH: 1:1 (6 mL)), calculated by GC.

**Table 2:**
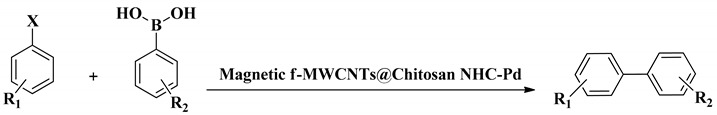
Suzuki reaction of various aryl halides with aryl boronic acids catalyzed by m-f-MWCNTs@chitosan NHC-Pd nanocomposite.

Entry	R_1_	R_2_	X	Amount of Catalyst (mol%)	Time (h)	Temperature (°C)	Conversion (%)	Yield (%)	TOF ^a^	TON
1	H	H	I	0.1	2	40	100	97	485	970
2	4-Me	H	I	0.1	2	40	100	97	485	970
3	4-OMe	H	I	0.1	2	40	100	97	485	970
4	H	H	Br	0.1	3	50	97	94	313	939
5	4-NO_2_	H	Br	0.1	3	50	98	95	316	948
6	4-Me	H	Br	0.1	3	50	94	91	303	909
7	H	H	Cl	1	5	50	N.R.	-	-	-
8	H	H	Cl	1	8	60	60	53	6.6	52.8
9	4-Me	H	Cl	1	8	60	53	50	6.25	50
10	4-NO_2_	H	Cl	1	8	60	68	62	7.75	62
11	H	Me	I	0.1	2	40	98	95	475	950
12	4-Me	Me	I	0.1	2	40	97	94	470	940
13	4-OMe	Me	I	0.1	2	40	96	93	465	930
14	H	Me	Br	0.1	3	50	92	89	296	888
15	4-Me	Me	Br	0.1	3	50	89	83	276	828
16	H	Me	Cl	1	8	60	55	50	6.25	50
17	4-Me	Me	Cl	1	8	60	50	47	5.87	46.96
18	4-OMe	Me	Cl	1	8	60	45	40	5	40

Reaction conditions: Aryl boronic acid (1.2 mmol), Aryl halide (1 mmol), K_2_CO_3_ 2–3 (mmol), m-f-MWCNTs@chitosan NHC-Pd nanocomposite as catalyst, Solvent (H_2_O-EtOH: 1:1 (6 mL)), calculated by GC. N.R. means no reaction. ^a^ TOF = Moles of desired product formed/moles of catalyst/time = TON/time.

**Table 3:** Comparison of the cross-coupling reaction results from m-f-MWCNTs@chitosan NHC-Pd nanocomposite with documented results from literature.

Entry	Catalyst (Pd Loading (mol%))	Reaction Conditions	Time (h)	Yield or Conversion (%)	Reusability	Ref.
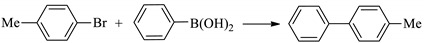						
1	poly(NIPAM-co-4-VP)-Pd (1)	K_2_CO_3_, H_2_O, 60 °C	5	95	5 times	[58]
2	GO-NH_2_-Pd(II) (1)	K_2_CO_3_, EtOH:H_2_O, 60 °C	4	71	10 times	[59]
3	GO-NHC-Pd(II) (0.25)	K_2_CO_3_, EtOH:H_2_O, 80 °C	20	94	6 times	[60]
4	rGO/Pd (0.5)	K_2_CO_3_, EtOH:H_2_O, 60 °C	1.5	71	6 times	[61]
5	m-f-MWCNTs@chitosan NHC-Pd nanocomposite (0.1)	K_2_CO_3_, EtOH:H_2_O, 50 °C	3	89	5 times.	This work
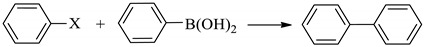						
6	Poly(NIPAM-4-VP AC)-Pd (0.05), X = Br	K_2_CO_3_, H_2_O, Ar atmosphere, 90 °C	1	92	6 times	[62]
7	GO-NH_2_-Pd(II) (1), X = Br	K_2_CO_3_, EtOH:H_2_O, 60 °C	4	73	10 times	[59]
8	Fe_3_O_4_@C-Pd@mSiO_2_ (1.5), X = I	K_2_CO_3_, iPrOH, 70 °C	6	92	6 times	[63]
9	Pd@Fe_3_O_4_@C (1), X = Br	K_2_CO_3_, DMF:H_2_O, 100 °C	4	99	-	[64]
10	m-f-MWCNTs@chitosan NHC-Pd (0.1), X = Br	K_2_CO_3_, EtOH:H_2_O, 50 °C	3	94	5 times	This work
